# Gadolinium-doped whitlockite/chitosan composite scaffolds with osteogenic activity for bone defect treatment: *In vitro* and *in vivo* evaluations

**DOI:** 10.3389/fbioe.2023.1071692

**Published:** 2023-02-15

**Authors:** Fei Xiao, Jingjing Shi, Xinhai Zhang, Min Hu, Kangming Chen, Chao Shen, Xiaodong Chen, Yaping Guo, Yang Li

**Affiliations:** ^1^ Department of Orthopedic Surgery, Xinhua Hospital Affiliated to Shanghai Jiaotong University, School of Medicine, Shanghai, China; ^2^ The Education Ministry Key Lab of Resource Chemistry and Shanghai Key Laboratory of Rare Earth Functional Materials, Shanghai Normal University, Shanghai, China; ^3^ Department of Orthopaedics, Huashan Hospital, Fudan University, Shanghai, China

**Keywords:** whitlockite, gadolinium, nanohybrid scaffolds, osteogenetic activity, GSK3β/β-catenin signaling pathway, bone defect disease

## Abstract

Reducing the incidence of bone defects caused by trauma and other primary diseases is an urgent task in modern society. In the present study, we developed a gadolinium-doped whitlockite/chitosan (Gd-WH/CS) scaffold and assessed its biocompatibility, osteoinductivity, and bone regeneration capacity for the treatment of calvarial defect in a Sprague-Dawley (SD) rat model. The Gd-WH/CS scaffolds possessed a macroporous structure, with a pore size ranging 200–300 μm, which facilitated the growth of bone precursor cells and tissues into scaffold. Results of cytological and histological biosafety experiments showed that both WH/CS and Gd-WH/CS scaffolds were non-cytotoxic to human adipose-derived stromal cells (hADSCs) and bone tissue, which demonstrated the excellent biocompatibility of Gd-WH/CS scaffolds. Results of western blotting and real-time PCR analysis provided a possible mechanism that Gd^3+^ ions in the Gd-WH/CS scaffolds promoted the osteogenic differentiation of hADSCs through the GSK3β/β-catenin signaling pathway and significantly upregulated the expression of osteogenic related genes (OCN, OSX and COL1A1). Finally, in animal experiments, SD rat cranial defects were effectively treated and repaired with Gd-WH/CS scaffolds due to its appropriate degradation rate and excellent osteogenic activity. This study suggests the potential utility of the Gd-WH/CS composite scaffolds in treating bone defect disease.

## 1 Introduction

In the 1990s, the incidences of bone defect and nonunion in fracture patients were approximately 5–10%; however, with the current aging society, the latest statistics indicate that the incidence has reached 1.9–20.0% (Mills et al., 2017). Therefore, reducing the incidences of bone defects caused by trauma, tumors, osteomyelitis, avascular necrosis surgical excision, and other primary diseases is an urgent task (Almubarak et al., 2016). The current standard treatment strategy is autograft transplantation (Karalashvili et al., 2018). However, the source of donor bone is limited, which hinders to repair large bone defects (Klifto et al., 2018). The present artificial bone substitutes clinically applied are costly and have insufficient osteogenic activity (Sun et al., 2017; Zhang et al., 2017). Furthermore, abnormal bone metabolism in osteoporosis and old patients makes their treatment even more difficult. Thus, materials scientists and orthopedics have attempted to develop novel bone tissue engineering scaffolds with osteogenic activity to promote bone regeneration post implantation, thereby preventing or treating bone defects (Balagangadharan et al., 2017; Jiang et al., 2018).

Natural bone is an organic/inorganic composite, comprising 25 wt% organic matrix, 65 wt% inorganic minerals, and 10 wt% water (Olszta et al., 2007). Whitlockite (WH, Ca_18_Mg_2_(HPO_4_)_2_(PO_4_)_12_) is an important inorganic component of bone tissues with a weight percentage of 20–35%, second only to hydroxyapatite [HA, Ca_10_(PO_4_)_6_(OH)_2_] (Shah, 2021). WH exhibits different degradability, osteoconductivity, and osteoinductivity than HA because of its distinct chemical composition and crystal structure (Kim et al., 2017; Hu et al., 2019). Especially in the early stage of bone regeneration, WH promotes cell proliferation and osteogenic differentiation and inhibits osteoclastogenesis (Kim et al., 2017; Cheng et al., 2018). Pouraghaei Sevari et al. suggested that the pro-osteogenic activity of WH is achieved *via* the activation of MAPK (mitogen-activated protein kinase) pathway (Pouraghaei Sevari et al., 2021). The highly concentrated Mg^2+^ and PO_4_^3–^ ions released from WH create a favorable microenvironment to inhibit the osteoclast differentiation from monocyte (Kim et al., 2017). During the bone remodeling process, the phase conversion from WH to HAP facilitates the formation of a dense neo-bone structure (Kim et al., 2017).

Chitosan (CS), a cationic polysaccharide-type biopolymer, originates from the shells of crustaceans (LogithKumar et al., 2016). Glycosaminoglycans (GAGs) are the main component necessary for proteoglycan synthesis in human articular cartilage and bone extracellular matrix (LogithKumar et al., 2016). In the context of biomaterials, the polysaccharide structure of CS is much similar to that of GAG, which endows CS with excellent biocompatibility and bioactivity (Tao et al., 2020). Although CS has bioactive characteristics, its poor mechanical properties limit its widespread clinical application (LogithKumar et al., 2016; Tao et al., 2020). Therefore, both WH and CS mimic the chemical composition of natural bone, which makes them have excellent bone histocompatibility, so the development of WH/CS composite scaffolds has great potential for bone tissue engineering application.

Rare Earth elements (REEs) have been used in ceramics, superconductors, nuclear reactor control rods, and magneto-optical materials. With the rapid development of rare Earth separation technology, its anti-inflammatory, bactericidal, analgesic, anti-cancer, anticoagulant, and other characteristics have been widely studied in the medical field (Barnewolt et al., 1997; Fricker, 2006; Zaichick et al., 2011). The physicochemical properties of rare Earth elements and calcium are similar. In order to prove that rare Earth elements may be involved in the regulation of bone metabolism, relevant studies have been conducted (Zhang et al., 2010). Evidence suggests that REEs show compound biological effects on bone formation and remodeling. For example, cerium and lanthanum promoted osteogenic differentiation of mesenchymal stem cells, while neodymium and samarium exhibited distinctive effects dependent on different concentrations (Wang et al., 2008; Zhang et al., 2010; Liu et al., 2013). As an REE, gadolinium (Gd)-based contrast agent has been widely used in magnetic resonance imaging. Studies have reported that Gd^3+^ stimulated DNA synthesis and induced calcium deposition in MC3T3-E1 cells in a dose-dependent manner *in vitro* (Quarles et al., 1994; Okada et al., 2011)*.* Another study showed that the [Gd@C_82_(OH)_22_]_n_ nanoparticles enhanced osteogenic activity *via* the BMP signaling pathway (Yang et al., 2013). The incorporation of Gd in magnesium alloys promoted the mechanical property of alloys (Kong et al., 2018). However, osteogenic activity and biosafety of Gd combined with WH remains unknown.

**SCHEME 1:**
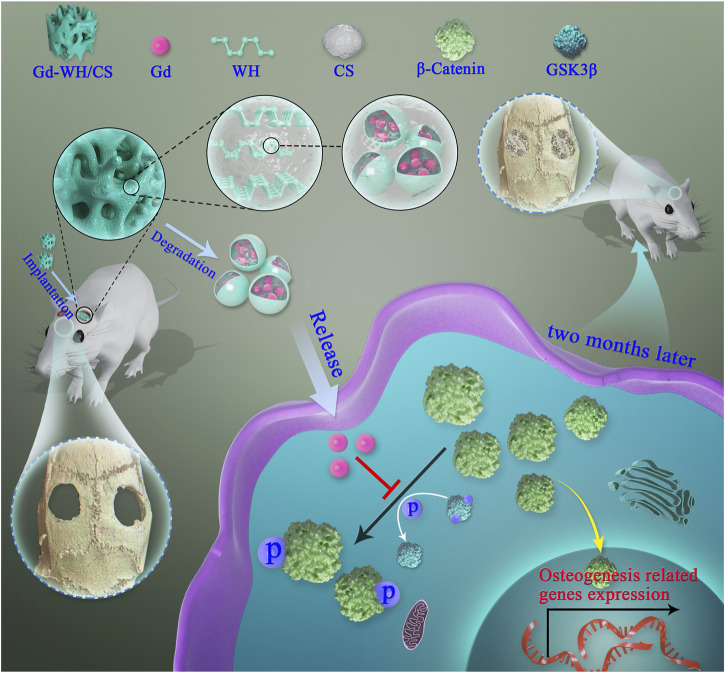
Illustration of Gd-WH/CS scaffolds promotion of osteogenic differentiation of hADSCs and growth of bone tissues *via* GSK3β/β-catenin signaling pathway.

In general, REEs exhibits beneficial biological effects at low concentrations, but may have negative effects at high doses (Pagano et al., 2015). To inhibit the toxicity caused by high Gd^3+^ concentration, developing Gd-doped WH nanoparticles is an alternative strategy. Based on the chemical composition and microstructures of natural bones, Gd-doped WH/CS composite scaffolds were constructed using a freeze-drying method for novel bone tissue engineering materials (Scheme 1). With these scaffolds, we determined whether the microenvironment would be conducive to cell adhesion, proliferation, and cell growth, and explored the effect of Gd^3+^ ion incorporation on osteogenic differentiation and bone regeneration performance of the nanocomposite scaffolds.

## 2 Materials and methods

### 2.1 Preparation of WH and Gd-doped WH

Pure WH particles were prepared by a high-temperature solid-state method. Calcium carbonate (CaCO_3_, 4.5041 g), magnesium hydroxide (Mg (OH)_2_, 0.2917 g), and diammonium hydrogen phosphate (NH_4_)_2_HPO_4,_ 4.6688 g were put in a ball mill and agitated for 4 h. The mixture was heated up to 350°C with a heating rate of 1°C/min and maintained at this temperature for 6 h. The obtained powders were mixed and heated up to 350°C for 30 min. Finally, the WH particles were obtained after calcination at 800°C for 8 h. Moreover, the Gd-doped WH particles were fabricated under the same conditions except that 5.0 mol% of Ca^2+^ ions were replaced by Gd^3+^ ions which is derived from Gd (NO_3_)_3_·6H_2_O (Aladdin Reagent network).

### 2.2 Preparation of WH/CS and Gd-doped WH/CS nanocomposite scaffolds

A homogeneous CS solution was obtained after 2.0 g of CS powder was dissolved in acetic solution (50 ml, 2.0 vol%). Then, 2.0 g of Gd-doped WH particles was added to the above CS solution at 20 ± 5°C (Zhou et al., 2017). The above mixtures were shifted to 24-well plates or 96-well plates. The specimen was frozen at −20°C, and then freeze-dried at −60°C for 80 h. The scaffolds were infused into 10.0wt% sodium hydroxide for 1 d. The samples were washed with deionized water until the pH value was neutral. In addition, the WH/CS nanocomposite scaffolds as a control group were prepared by the same method.

### 2.3 Isolation and expansion of hADSCs

All patient consent and experimental protocols were reviewed and approved by the Institutional Ethics Committee of Xin Hua Hospital Affiliated to Shanghai Jiao Tong University School of Medicine.

Human adipose-derived stem cells (hADSCs) were donated by 5 female patients aged 33.5 years (range, 30–36 years) who accepted subcutaneous liposuction surgery. Informed consent was obtained from all donors. None of the patients presented with osteoporosis, other orthopedic diseases or systemic diseases. The isolation operation process was according to a reported protocol (Estes et al., 2010). Briefly, adipose tissue was finely minced and digested in a 0.1% collagenase solution (Sigma) and incubated at 37°C with vigorous shaking for 30 min. After digestion, the tissue was centrifuged, and the pellet was resuspended in phosphate buffer saline (PBS). Then, the cell suspension was filtered through 100 μm and 40 μm nylon mesh cell strainers to remove undigested fat tissue and then inoculated in a 10 cm dish containing a culture medium (CM) which contained low-glucose (1 g l^−1^) Dulbecco’s modified Eagle’s medium (DMEM; Gibco), 10% fetal bovine serum (FBS; Gibco), 100 U ml^−1^ penicillin, and 100 μg ml^−1^ streptomycin (Hyclone). The CM was replaced every 2 d. hADSCs between passages 3 and 5 were used for subsequent experiments.

### 2.4 Osteogenic differentiation of hADSCs

For osteogenic differentiation assays, hADSCs were seeded and cultured in CM until confluent. After that, the cells were induced by normal OM, which was mixed with CM and 100 nM dexamethasone, 50 μM L-ascorbic acid, and 10 mM β-sodium glycerophosphate. On day 7, the cells were fixed in 4% paraformaldehyde (PFA) for 10 min, and ALP staining was performed according to the manufacturer’s instructions (Rainbow). On day 14, after fixation in 4% PFA, the cells were incubated with 40 mM Alizarin Red S (ARS) staining solution (Sigma) for 20 min at room temperature (20–26°C). A semi-quantitative analysis of ALP activity and ARS staining were performed as previously described (Zhang et al., 2013). Briefly, after the cells were lysed, the total protein content of the samples was determined using a BCA Protein Assay kit (Thermo Fisher). ALP activity was detected at 405 nm after 30 min incubation at 37°C using p-nitrophenyl phosphate (p-NPP) (Sigma) as the substrate. After the ARS staining, 10% cetylpyridinium chloride (Sigma) was added to dissolve extracellular calcium deposits for 1 h; the dissolved solution was distributed at 100 μl per well on a 96-well plate, and absorbance readings were taken at 590 nm using a microplate reader (Tecan). The ALP activity and ARS levels were normalized to the total protein content (absorbance index). Experiments were repeated independently three times.

### 2.5 Western blotting

Western blotting was performed as previously described (Xiao et al., 2018). After stimulation by OM with or without various Gd ion concentrations, the cells were harvested, and the total proteins were extracted using RIPA buffer (Beyotime). After separated by 10% or 8% SDS-PAGE, lysate proteins were transferred to polyvinylidene difluoride (PVDF) membranes. The PVDF membranes were then blocked with 5% (w/v)bovine serum albumin (BSA) for 1 h and incubated with primary antibodies diluted in 1% (w/v) BSA in TBS-Tween overnight at 4°C. Horseradish peroxidase-labeled secondary antibodies were added and visualized using an enhanced chemiluminescence detection system (Millipore, United States). Experiments were repeated independently three times.

### 2.6 Real-time PCR analysis

Total RNA was extracted using the TRIzol reagent and reverse transcribed using the Prime Script RT Master Mix cDNA Synthesis Kit (TaKaRa) to obtain first-strand cDNA. Real-time PCR reactions were performed on the VIIA7 instrument (Applied Biosystems) using the SYBR Green PCR Kit (TaKaRa). The real-time PCR conditions were set as follows: denaturation at 95°C for 30 s, followed by 45 cycles of 95°C for 10 s and 60°C for 30 s. The sequences of the gene primers used are as follows: OCN, forward: 5′-GAA​GCC​CAG​CGG​TGC​A-3′ and reverse: 5′-CAC​TAC​CTC​GCT​GCC​CTC​C-3'; OSX, forward: 5′-CCT​CTG​CGG​GAC​TCA​ACA​AC-3′ and reverse: 5′-AGC​CCA​TTA​GTG​CTT​GTA​AAG​G-3'; COL1A1, forward: 5′-GAG​GGC​CAA​GAC​GAA​GAC​ATC-3′ and reverse: 5′-CAC​TAC​CTC​GCT​GCC​CTC​C-3′ and GAPDH, forward: 5′-ACA​ACT​TTG​GTA​TCG​TGG​AAG​G-3′ and reverse: 5′-GCC​ATC​ACG​CCA​CAG​TTT​C-3'. All quantitation was normalized to an endogenous control, GAPDH, and the data were analyzed using the 2^−ΔΔCT^ method. Experiments were repeated independently three times.

### 2.7 Preparation of scaffolds extracts

The WH/CS and Gd-WH/CS scaffolds were prepared as small slices with a thickness of 3 mm and a diameter of 15 mm for *in vitro* experiments, and these scaffold slices were sterilized under ultraviolet light for 24 h.

For the preparation of scaffold extracts, 1g of scaffold samples were soaked in 3 ml of low-glucose DMEM and incubated at 37°C. Then, the medium was filtered through a 0.22 mm filter (Millipore, United States) to remove the particulates after 48 h. The extracts, supplemented with 10% FBS, 10 mM β-sodium glycerophosphate, 100 nM dexamethasone, 50 μM L-ascorbic acid, 100 μg ml^−1^ streptomycin and 100 U ml^−1^ penicillin were mixed as WH/CS extract osteogenic medium (WH/CS_ext_-OM) and Gd-WH/CS extract osteogenic medium (Gd-WH/CS_ext_-OM). Experiments were repeated independently three times.

### 2.8 Live/dead assay and proliferation assay

Cell viability was detected by a Live/Dead assay kit (Invitrogen). Briefly, after incubation of hADSCs on WH/CS and Gd-WH/CS scaffolds at a density of 2.5×10^4^ cells cm^−2^ for 2 days, the ethidium homodimer-1 and calcein were added for 15 min at 37°C. The cells were observed using confocal microscopy. Red and green fluorescence signals respectively represent dead and live cells. Cell proliferation was assessed by Cell Counting Kit-8 (CCK-8; Dojindo Molecular Technologies, Inc.). The hADSCs were seeded on WH/CS and Gd-WH/CS scaffolds and cultured for 1, 3, 5, and 7 days in CM. After culturing for the indicated time, the DMEM and CCK-8 solution mixture were added. The optical density (OD) was detected using a microplate reader (Tecan) at 450 nm (650 nm as reference). The effect of Gd ion on hADSCs proliferation was detected according to the above method.

### 2.9 Attachment and morphology assay

For the cell morphology analysis, hADSCs were cultured on the WH/CS and Gd-WH/CS scaffolds in 24-well plates for 3 days. After culture, the cell-seeded samples were softly rinsed with PBS twice, fixed with 2.5% glutaraldehyde in PBS overnight, and dehydrated in a graded series of ethanol (30, 50, 75, 85, 95, and 100%). Then, the specimens were dried for 4 h at 37°C, coated with gold, and examined using SEM.

### 2.10 Animal experiments

All surgical procedures were performed on 12-week-old female Sprague-Dawley rats as previously described (Chen et al., 2017). Twenty rats were randomly allocated into the following graft study groups: WH/CS group and Gd-WH/CS group. The scaffolds were trimmed into 2 mm and 5 mm in thickness and diameter (weight: 0.3g), respectively. A 1.0–1.5 cm sagittal incision was made on the scalp and two full-thickness critical-sized calvarial defects were created by means of a 5-mm diameter electric trephine. The scaffolds were implanted into the defect regions. Then, the wounds were sutured with 4–0 silk sutures. After surgery, each rat received intramuscular injection of antibiotics and all the animals were allowed free access to food and water.

### 2.11 Sequential fluorescent labeling

A sequential fluorescent labeling was performed to detect the mineralization of new bone tissue as previous report (Ding et al., 2017). At 2-, 4-, and 6-weeks post operation, the rats were subjected to an intraperitoneal injection of different fluorochromes, as follows: calcein (5 ml kg^−1^ of body weight), Alizarin Red (0.8 ml kg^−1^ of body weight), and tetracycline (1 mg kg^−1^ of body weight).

### 2.12 Micro-CT scanning

After 8 weeks of postoperation, the animals were euthanized and the skulls were collected and fixed by 4% PFA for 24 h and scanned by micro-CT at 18-μm resolutions. After scan, the visualization of the bone was software-generated. The percentage of newly formed bone volume relative to tissue volume (BV/TV) and the bone mineral density (BMD) was determined using the analysis software.

### 2.13 Histomorphometric analysis and histological observations

After dehydration, specimens were embedded in polymethylmethacrylate and sliced to a final thickness of 40 μm, as described previously (Ding et al., 2017). The fluorescent labeling was observed by a laser confocal microscope (ZEISS). In addition, sections were stained with van Gibson’s picrofuchsin. Yellow and Red indicated scaffolds and newly formed bone, respectively. The surface areas of the new bone tissue were measured using Image Pro 5.0. The other specimens were embedded in paraffin after decalcified by 12.5% EDTA and stained with hematoxylin and eosin (H&E). For immunohistochemical staining, paraffin sections were deparaffinated, and the sections were incubated with osterix and osteocalcin overnight at 4°C. Secondary antibodies and DAB substrate were used to stain the sections. The images were captured using a Nikon Eclipse E600 microscope.

### 2.14 Statistical analysis

Data are presented as the mean ± standard deviation (SD) and analyzed using an unpaired Student’s *t*-test in SPSS 19.0. *p* < 0.05 was considered a statistically significant.

## 3 Results

### 3.1 Structure and morphology of WH and Gd-doped WH powders

**FIGURE 1:**
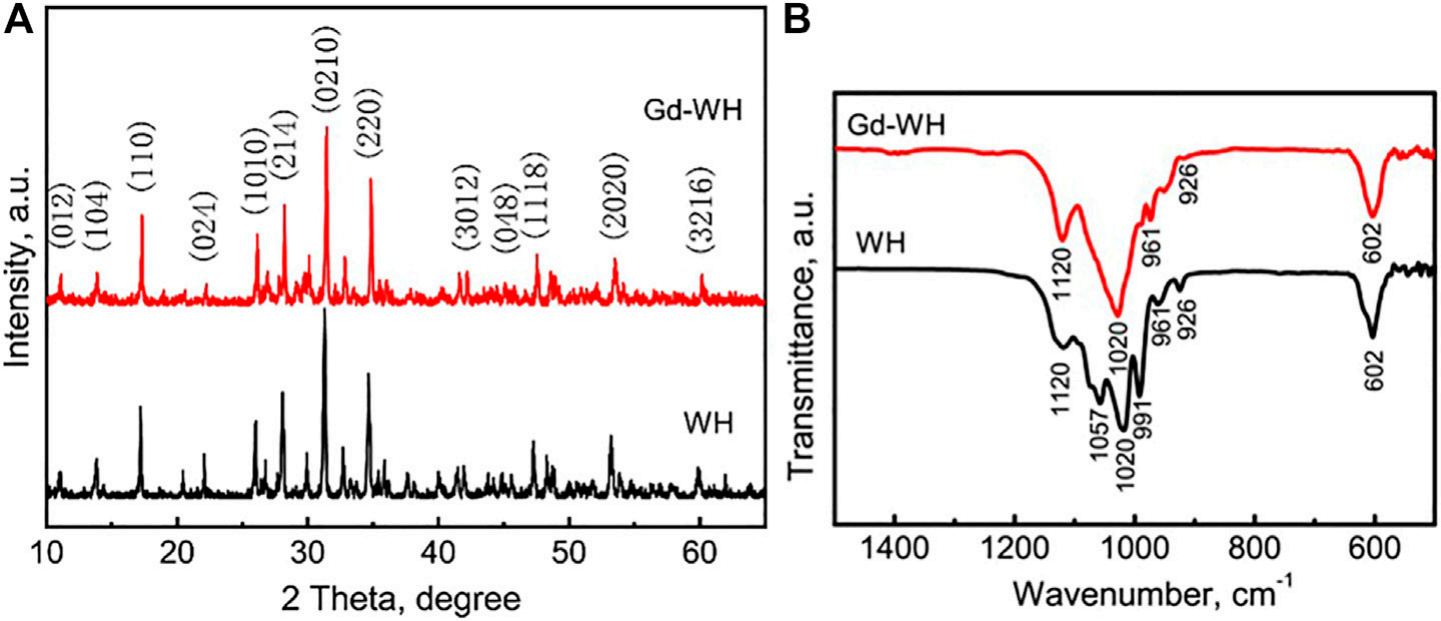
**(A)** XRD patterns and **(B)** FTIR spectra of WH and Gd-doped WH powders.

WH and Gd-doped WH powders were synthesized by a high-temperature solid-state reaction. As shown in Figure 1A, the XRD pattern of the WH particles had characteristic peaks of a rhombohedral (R3c) structure (JCPDS card no. 70–2064). The XRD pattern of the Gd-WH particles had diffraction peaks similar to those of the pure WH particles, suggesting that the incorporation of Gd^3+^ ions did not affect the phase structures (Figure 1). Moreover, the Gd^3+^ dopants had no significant effects on the lattice constants, as confirmed by the same diffraction peak positions in the XRD spectra of the Gd-WH and WH particles. This was attributed to the possibility that the ionic radii of Gd^3+^ ions (93.8 pm) and Ca^2+^ ions (99 pm) are close. Moreover, the functional groups of pure WH and Gd-doped WH particles were detected by FTIR spectra (Figure 1B). The characteristic peaks in the range from ν_max_/cm^−1^ 991 to 1,057 were ascribed to the *v*_3_ vibration of PO_4_^3−^ ions (Hu et al., 2019; Kaliannagounder, et al., 2021). The peaks due to *v*_4_ bending vibration of the O–P–O bond and *v*_1_ stretching vibration of the P‒O are located at ν_max_/cm^−1^ 602 and 962, respectively (Hu et al., 2019; Kaliannagounder, et al., 2021). The peak at ν_max_/cm^−1^ 1,120 corresponded to the asymmetric stretching vibration of the PO_4_^3–^ group. In addition, the P‒O‒H stretching peak due to HPO_4_^2−^ ions was detected at ν_max_/cm^−1^ 921, suggesting that the HPO_4_^2−^ ions existed in both the WH and Gd-WH particles (Hu et al., 2019; Kaliannagounder, et al., 2021).

**FIGURE 2:**
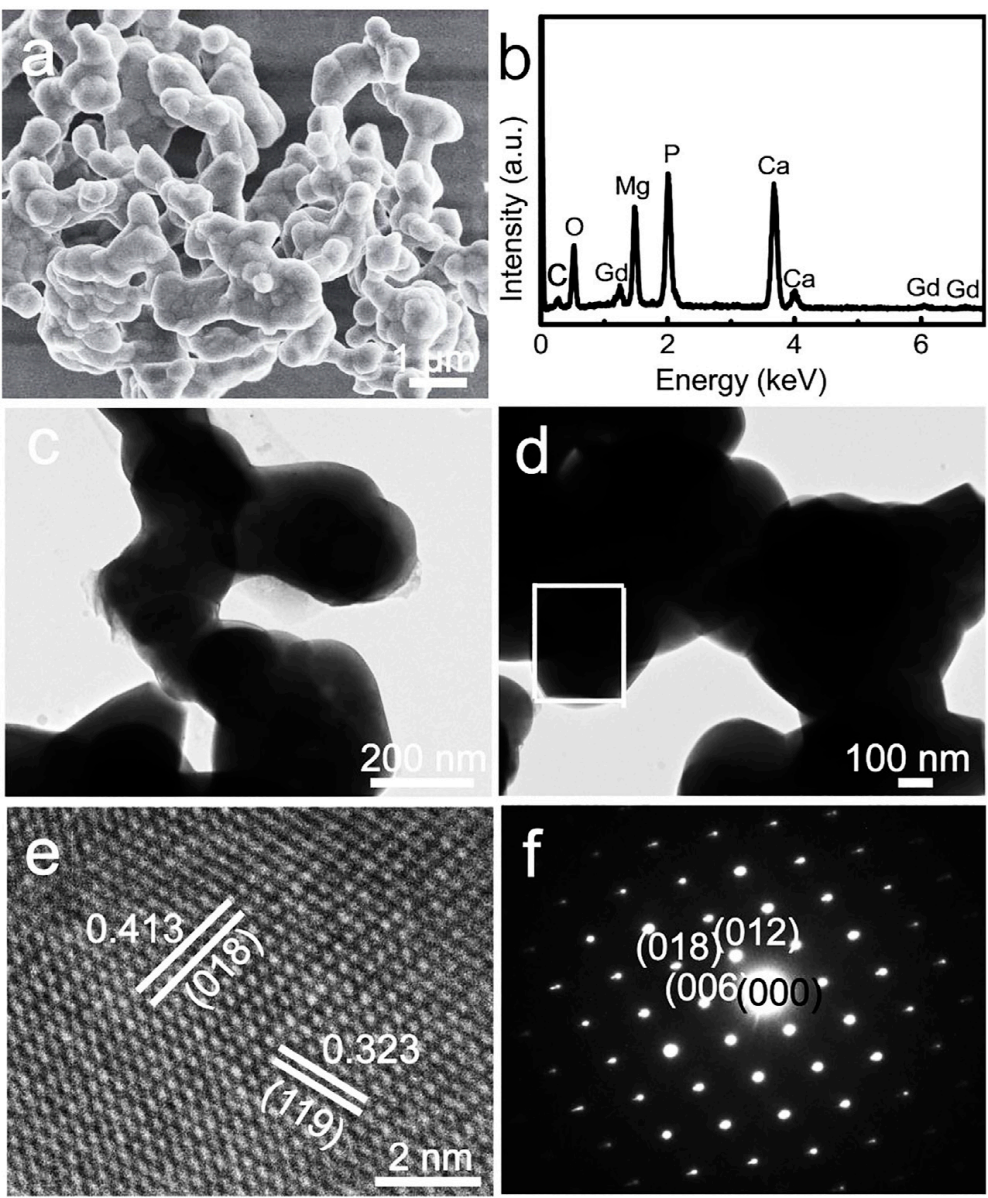
**(A)** SEM image, **(B)** EDS spectrum, **(C, D)** low-resolution TEM images, **(E)** high-resolution TEM image, and **(F)** SAED pattern of Gd-WH particles.

The scanning electron microscopy (SEM) images indicate that the Gd-WH particles tend to aggregate during the high-temperature solid-state reaction process (Figure 2A). These Gd-WH particles showed irregular shapes and a wide particle size distribution between 200 and 300 nm, which were further confirmed by the low-resolution TEM images (Figures 2C, D). The EDS spectrum in Figure 2B indicates that the Gd-WH particles consisted of Mg, Ca, P, O, and Ga elements, suggesting the incorporation of Gd dopants. The presence of carbon originated from the conducting resin in the SEM characterization process. The high-resolution TEM images show significant lattice fringes (Figure 2E). Furthermore, the d-spacings of 0.413 nm and 0.323 nm corresponded to the (018) and (119) planes of Gd-WH crystals, respectively. The SAED pattern also demonstrated that the Gd-particles exhibited a monocrystalline structure with a [100] zone axis (Figure 2F).

### 3.2 Structure and morphology of WH and Gd-doped WH scaffolds

**FIGURE 3:**
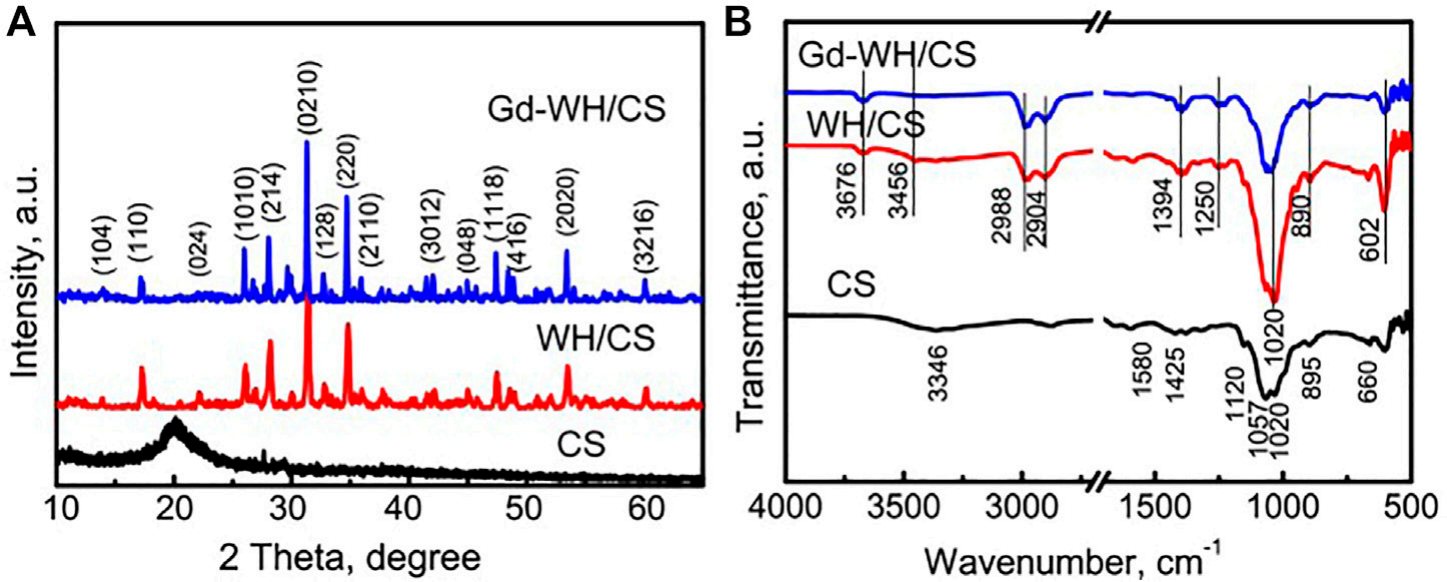
**(A)** XRD patterns of the CS powders, WH/CS scaffolds, and Gd-WH/CS scaffolds; **(B)** FTIR patterns of the CS powders, WH/CS scaffolds, and Gd-WH/CS scaffolds.

The phases of the CS powders, WH/CS scaffolds, and Gd-WH/CS scaffolds were detected *via* XRD (Figure 3A). As shown in the XRD pattern of the CS powders, the broad peak at 20.5° and two weak peaks at 10.8° and 28.5° demonstrated that the CS organic matrix had a semi-crystalline feature (Chen et al., 2022). After the combination of the CS organic matrices with WH or Gd-WH particles, only the characteristic peaks of the WH phase were observed in the XRD patterns of the WH/CS and Gd-WH/CS scaffolds (Figure 3A). The absence of the CS characteristic peaks in the composite scaffolds may be attributed to its low crystallinity.

The functional groups of the scaffolds were further listed by FTIR (Figure 3B). It was found that the prepared scaffolds were PO_4_^3-^ in the range of 1,020–1,057 cm^−1^ $v_{3}$ vibration peak, C-O stretching vibration absorption peak in the CS. The stretching vibration peaks of P-(OH) are at 890–895 cm^−1^, The bending vibration peak ( $v_{4}$ ) of O-P-O bond of PO_4_^3-^ was 602 cm^−1^ and asymmetric tensile vibration peak of PO_4_^3-^ was found at 1,120 cm^−1^ (Jang et al., 2014; Kim et al., 2017). The absorption peak at 1,394 cm^−1^ can be attributed to the vibrational absorption peak of the amide group in CS. The absorption peak at 1,580 cm^−1^ is due to the amino group vibration in CS. The presence of absorption peaks at 3,346 cm^−1^ and 3,456 cm^−1^ is due to the presence of hydroxyl groups.

**FIGURE 4:**
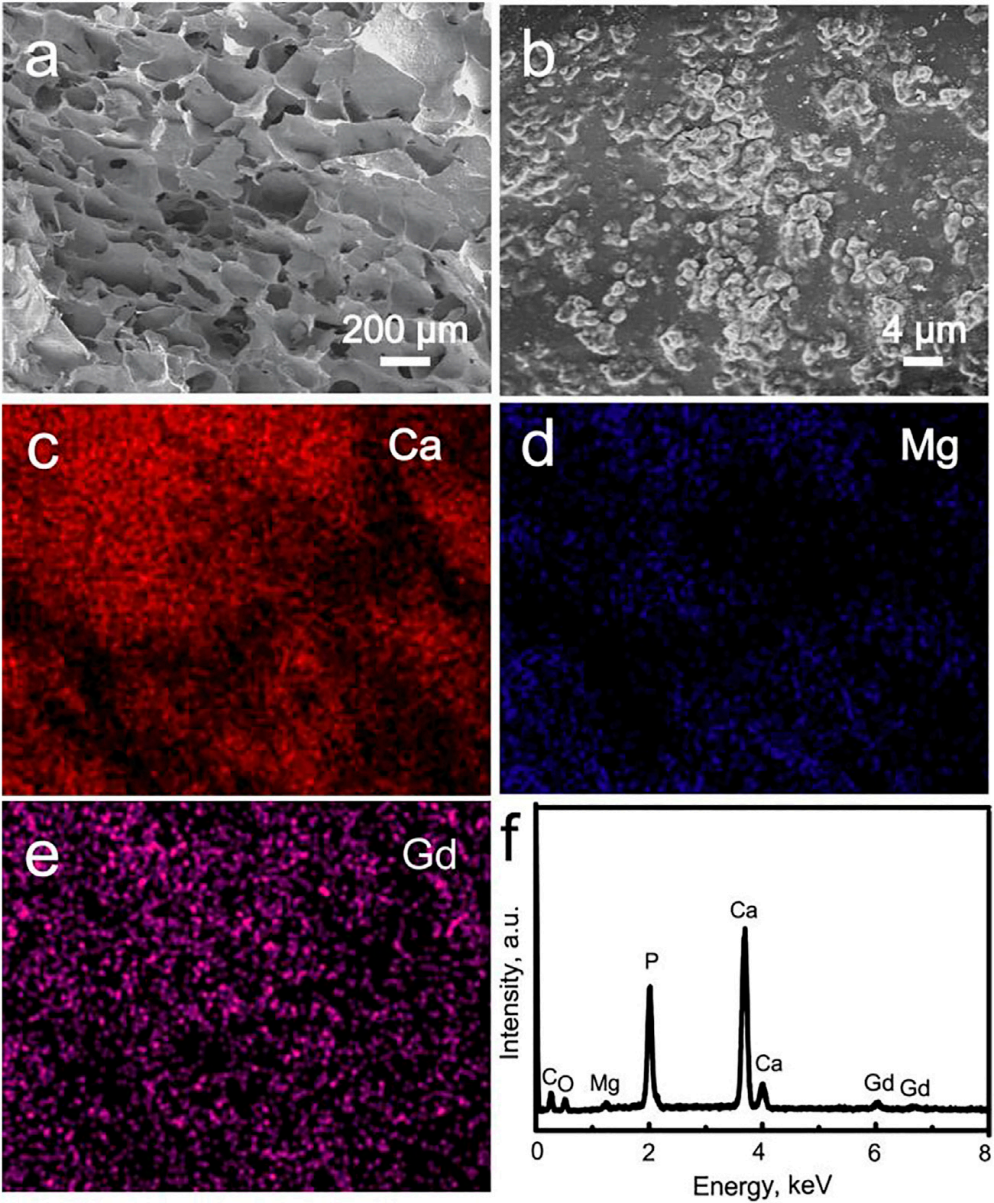
**(A, B)** SEM images of the Gd-WH scaffolds; element distribution images: **(C)** Ca, **(D)** Mg, and **(E)** Gd; **(F)** EDS spectrum acquired from the image b.

**FIGURE 5:**
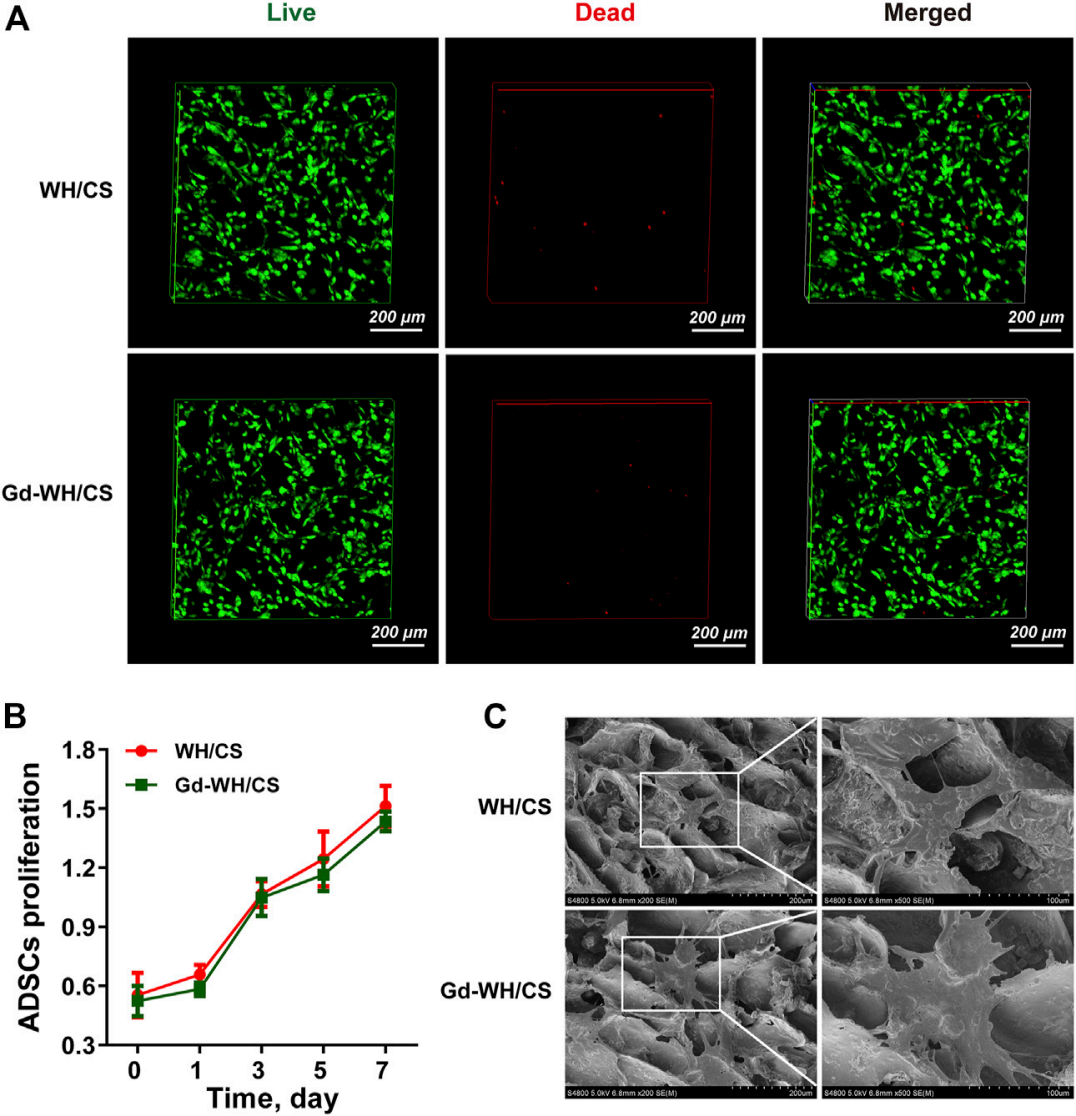
Cytocompatibility of Gd-WH/CS scaffolds with hADSCs growth. **(A)** Live/dead cell staining of hADSCs on WH/CS and Gd-WH/CS scaffolds after 3 days of culture. **(B)** Proliferation of hADSCs cultured on different scaffolds. **(C)** Representative SEM images of hADSCs seeded on the indicated scaffolds after 3 days of culture.

The morphology and elemental composition of the Gd-WH/CS composite scaffolds were characterized by the SEM images and corresponding EDS patterns, respectively (Figure 4). The low-resolution SEM image indicated that the Gd-WH/CS composite scaffolds possessed 3D interconnected macropores with a pore size of approximately 200–300 μm (Figure 4A). These macropores were created *via* the sublimation of ice crystals in the freeze-drying procedure. (Hu et al., 2019). The Gd-WH/CS composite scaffolds were constructed by the CS films, and the Gd-WH particles were embedded in the organic matrices (Figure 4B). The Ca, Mg, and Ce distribution images further demonstrated that the Gd-WH particles were dispersed in the whole CS films (Figures 4C, E). The EDS spectrum in Figure 4F showed that the Gd-WH/CS composite scaffolds comprised Mg, Gd, Ca, P, C, and O (Figure 5F). The Mg, Gd, Ca, and P elements originated from the Gd-WH particles, whereas C was derived from CS.

### 3.3 Cytocompatibility of Gd-WH/CS scaffolds with hADSCs growth

hADSCs are considered as promising seed cells in bone tissue engineering (Correia et al., 2012). Excellent cell compatibility is the most important prerequisites for scaffold to ensure stem cell adhesion, spreading and proliferation. Figure 5A shows only a few dead cells (stained red) in all cultures grown on both WH/CS and Gd-WH/CS scaffolds, indicating that all scaffolds were safe for hADSC growth.

In addition, the results of CCK-8 assay showed an increasing number of hADSCs on the scaffolds over time, confirming good biocompatibility of the WH/CS scaffolds. There is no significant difference in hADSCs number was observed among the WH/CS and Gd-WH/CS scaffolds (Figure 5B), suggesting that neither of the two scaffolds were toxic to hADSCs. In order to directly observe the morphology of hADSCs on different scaffolds, SEM images of cells adhering to scaffolds were taken 3 days after cell seeding. The hADSCs spread on WH/CS and Gd-WH/CS scaffolds, showing clear polygona shape and random orientations. (Figure 5C). Taken together, WH/CS and Gd-WH/CS scaffolds were nontoxic and supported the proliferation of hADSCs.

### 3.4 Effects of Gd-WH/CS extracts on osteogenic differentiation of hADSCs

**FIGURE 6:**
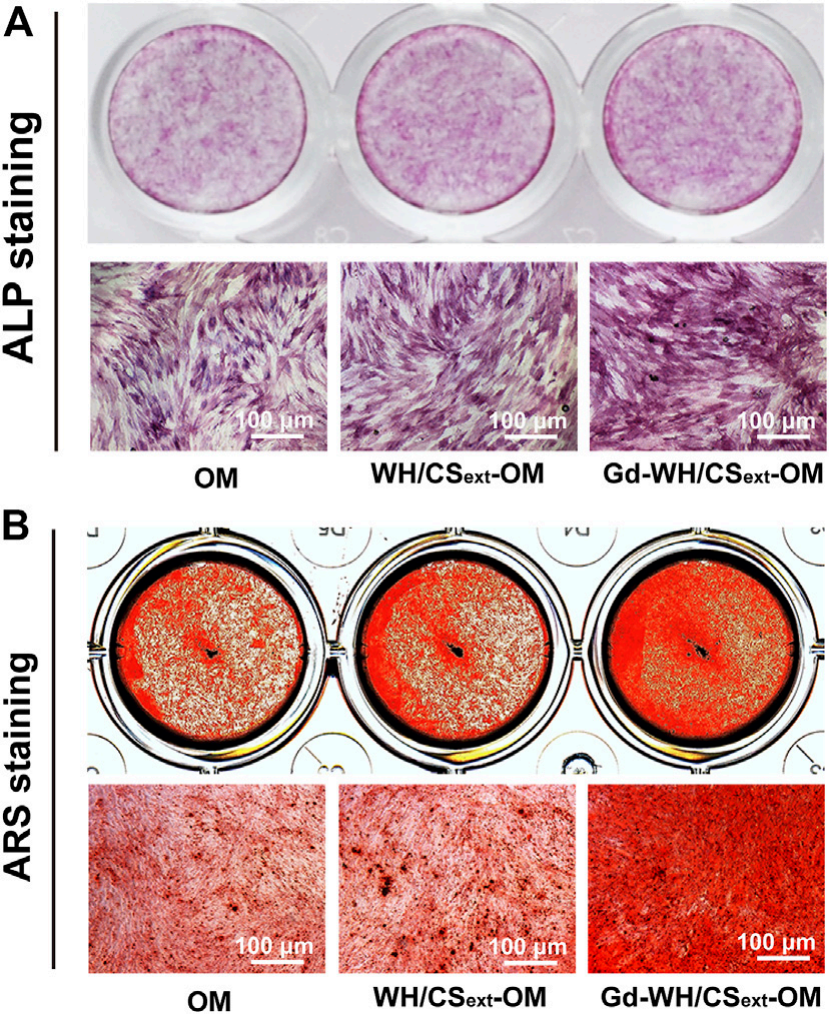
ALP activity and matrix mineralization of hADSCs treated with different medium. **(A)** ALP staining of hADSCs after 7 days of osteogenic induction. **(B)** ARS staining of hADSCs after 14 days of osteogenic induction.

To investigate the effects of WH/CS and Gd-WH/CS extracts on osteogenic differentiation, hADSCs were treated with OM, WH/CS_ext_-OM, or Gd-WH/CS_ext_-OM during osteoclast formation. ALP activity can be considered an early biomarker for osteogenic differentiation (Liu et al., 2013). In this study, ALP staining showed that Gd-WH/CS_ext_-OM treated hADSCs showed significantly higher ALP activity than OM after 7 days of culture (Figure 6A). However, there was no significant difference in ALP activity among the hADSCs treated with WH/CS_ext_-OM and OM (Figure 6A). Furthermore, we detected the matrix mineralization by ARS assays of hADSCs treated with different media after osteogenic induction for 14 days. Calcium deposition was higher in the Gd-WH/CS_ext_-OM group than in the WH/CS_ext_-OM and OM groups (Figure 6B), and the results were consistent with the results of ALP staining.

### 3.5 Effects of Gd on the osteogenic differentiation of hADSCs

**FIGURE 7:**
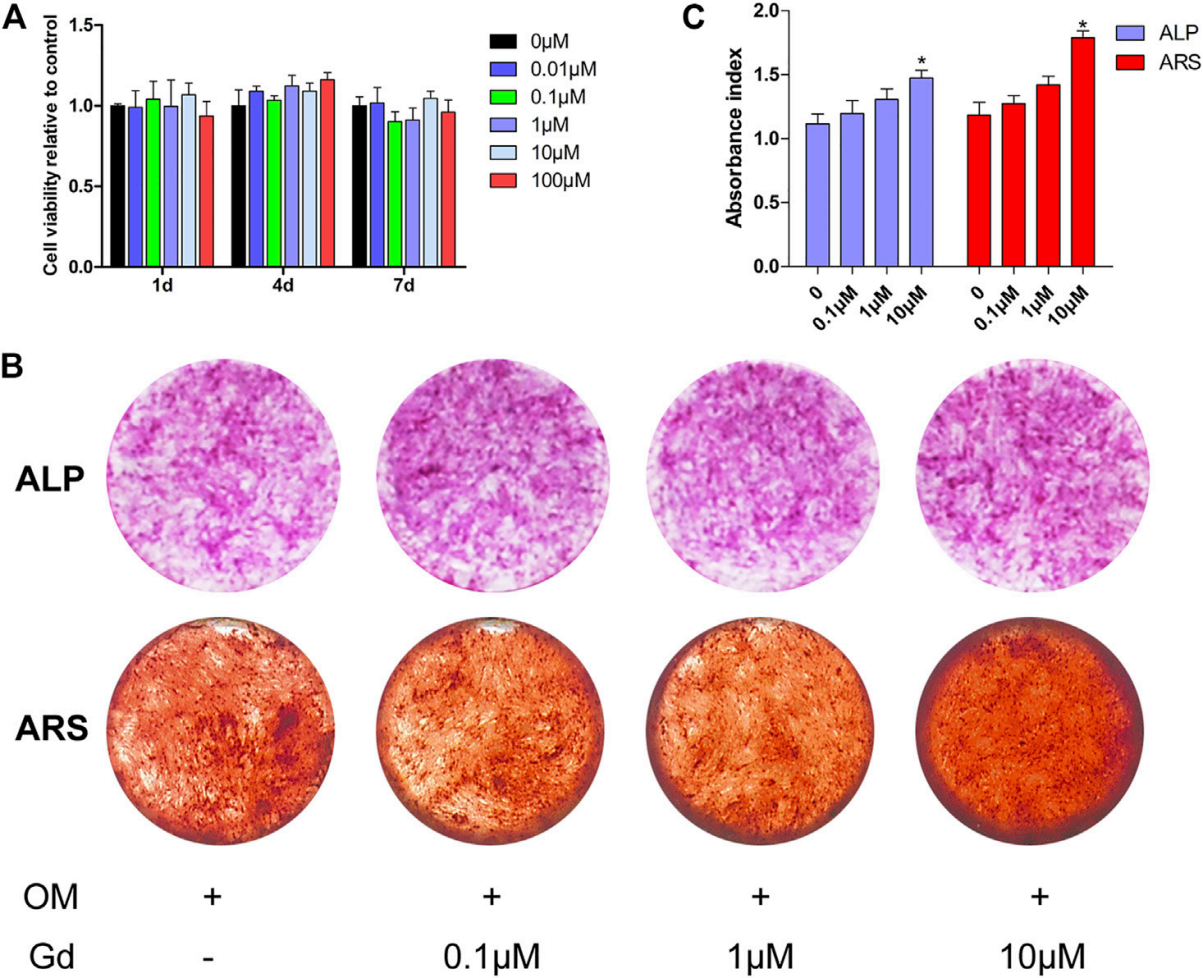
ALP activity and ARS staining of hADSCs treated with Gd during osteogenic induction. **(A)** CCK-8 assay measured cell viability. **(B)** ALP and ARS staining of hADSCs following osteogenic induction. **(C)** Quantification of ALP activity and extracted ARS; *p < 0.05.

To investigate the effect of Gd on osteogenic differentiation, hADSCs were treated using an osteogenic medium (OM) in different Gd ion concentrations. No cytotoxic effect of Gd on hADSCs was detected at the indicated dose (Figure 7A). The staining showed that hADSCs treated with OM supplemented with 10 μΜ Gd exhibited slightly higher ALP staining after 7 days of osteogenic induction and more calcium deposition after 14 days of culture than those of OM (Figure 7B); the results from the quantification of ALP activity and extracted ARS were consistent with the results of staining (Figure 7C).

### 3.6 Gd enhanced osteogenic differentiation *via* GSK3β/β-catenin signaling pathway

**FIGURE 8:**
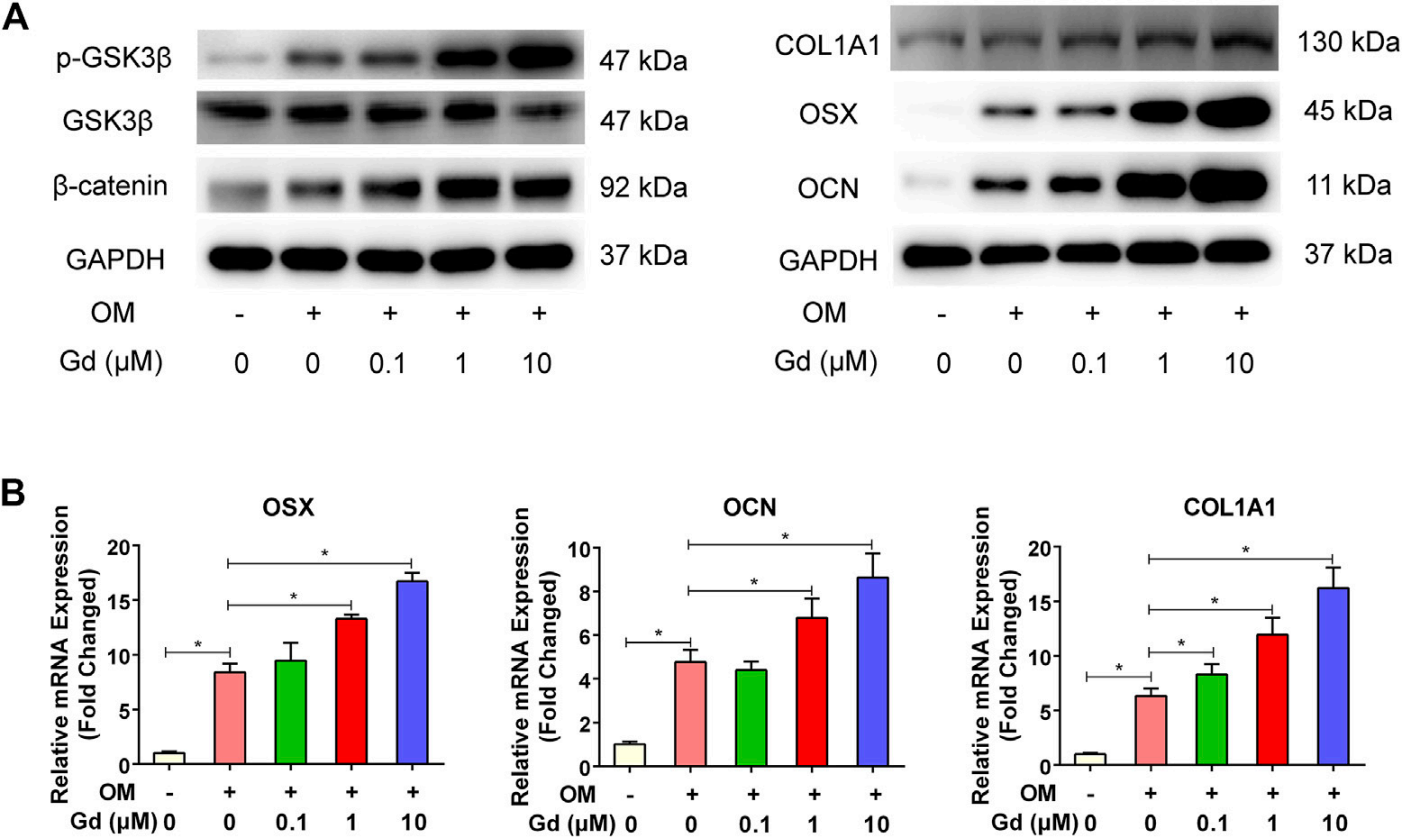
Gd enhanced osteogenic differentiation *via* GSK3β/β-catenin signaling pathway. **(A)** Left: Immunoblot of p-GSK3β and β-catenin indicated that Gd increased the phosphorylation level of GSK3β and accumulation of β-catenin after OM stimulation for 4 h; Right: Gd increased the expression of COL1A1, OSX, and OCN proteins after osteogenic induction for 7 days. **(B)** The relative mRNA of hADSCs treated with Gd during osteogenic induction for 7 days; *p < 0.05.

The Wnt signaling pathway plays a critical role in regulating osteogenic differentiation. The activity of canonical Wnt signaling is mediated through GSK3β, which promotes phosphorylation of β-catenin and ultimately results in the degradation of β-catenin and initiates the transcription of osteogenic-related genes in cell nuclei (Liu et al., 2014; Zhang et al., 2016). Thus, inhibition of GSK3β promotes osteogenic differentiation. Hence, we assessed whether enhancing osteogenic differentiation by Gd was associated with inhibition of GSK3β activity. Western blot results demonstrated that Gd decreased the GSK3β and increased the β-catenin activity in a dose-dependent manner (Figure 8A). Furthermore, the expression of osteogenic-related genes, including OCN, OSX, and COL11A were quantified using real-time PCR. The results show that the expression of these genes increased with the treatment of Gd in a dose-dependent manner (Figure 8B).

### 3.7 Gd-WH/CS scaffold promotes bone regeneration *in vivo*

**FIGURE 9:**
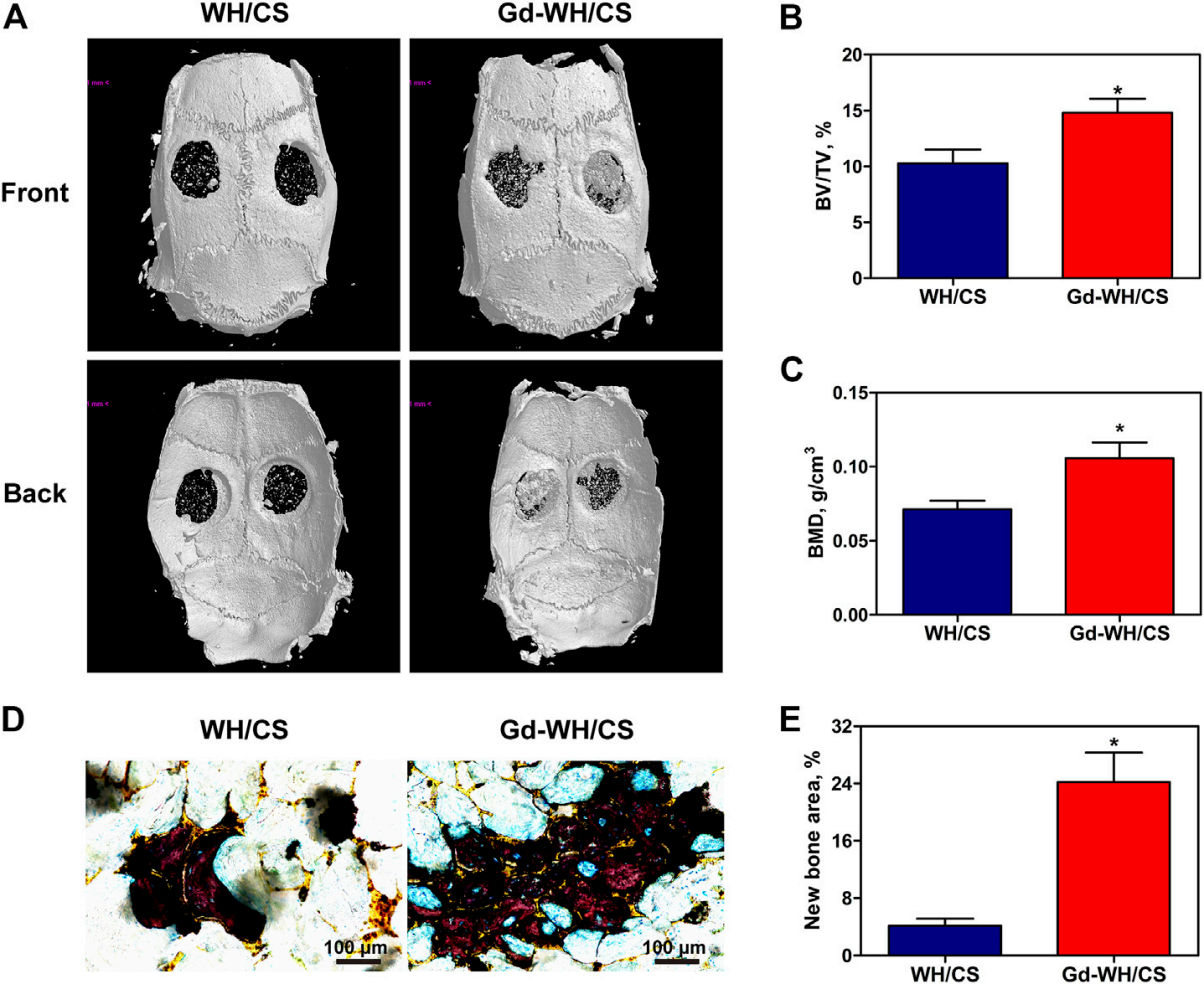
Micro-CT evaluation and van Gieson staining of calvarial bone repair. **(A)** Representative 3-D front and back images of calvarial bone defects taken at 8 weeks post-operation. **(B)** Morphometric analysis of bone volume/tissue volume (BV/TV) ratio. **(C)** Morphometric analysis of BMD. **(D)** Histological images of newly formed bone in calvarial defects. **(E)** The percentage of new bone area assessed using histomorphometric analysis; *p < 0.05.

In order to investigate whether Gd-WH/CS scaffold can play a role in promoting bone repair *in vivo,* critical-sized bone defects model was established and the formation of new bone was quantified by micro-CT. As shown in Figure 9A, the calvarial bone defects treated with Gd-WH/CS revealed more new bone formation than WH/CS. The BV/TV for the Gd-WH/CS and WH/CS groups arrived at 14.81 ± 3.07% and 10.30 ± 2.98%, respectively (Figure 9B), and the BMD showed the same pattern as the BV/TV (Figure 9C). These results suggested that Gd-WH/CS scaffolds possess stronger enhancing effects on bone regeneration than WH/CS scaffolds. Van Gieson staining further supported the findings of micro-CT.-Stained tissue in the scaffold possessed clear trabecular structure, representing newly formed bone. As shown in Figures 9D, E, there were few newly formed bone tissues in WH/CS group, while more new bone tissues were found in Gd-WH/CS group.

### 3.8 Fluorochrome labeling histomorphometric analysis

**FIGURE 10:**
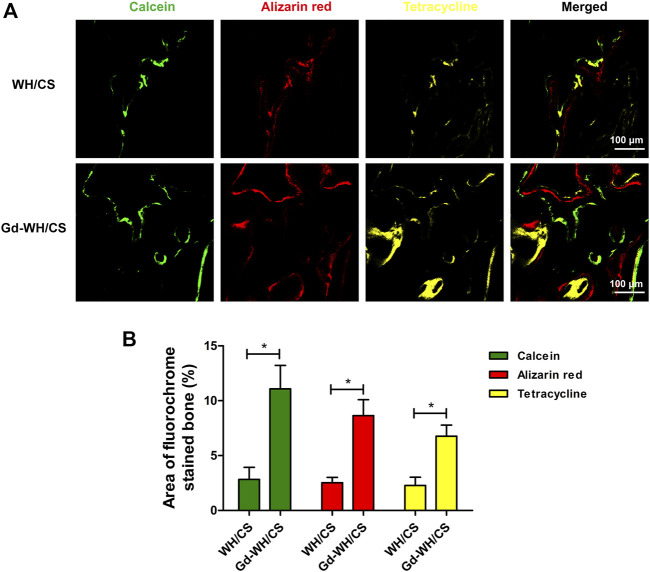
New bone formation and mineralization determined histomorphometrically using fluorochrome-labeling analysis. **(A)** Confocal microscope images for each group. Row 1 (green) represents new bone formation labeled *via* calcein injected at week 2; row 2 (red) represents Alizarin Red at week 4; row 3 (yellow) represents tetracycline at week 6; row 4 represents merged images of the three fluorochromes for the same group. **(B)** The graph shows the percentages of each fluorochrome area for different groups; *p < 0.05.

In this study, the mineralization of new bone was labeled by calcein, alizarin red, and tetracycline fluorescence. As shown in Figure 10, the different fluorescent labels representing new bone mineralization were observed 2–6 weeks post operation. After 2 weeks, calcein was deposited onto the Gd-WH/CS scaffolds in a broader area than in WH/CS scaffolds, exhibiting more intense and extensive green fluorescence. The percentages of calcein-labeled areas in the Gd-WH/CS and WH/CS groups reached 11.09 ± 2.83% and 2.13 ± 1.11%, respectively. Furthermore, Alizarin Red (4 weeks) was barely found in WH/CS scaffolds, while the percentages of Alizarin Red labeled areas in the Ce-WH/CS group was 8.64 ± 2.53%. At 6 weeks, tetracycline absorbed into the bone area manifested more intensity and extensity in Gd-WH/CS group (6.77 ± 2.28%) than in WH/CS (1.02 ± 0.76%), indicating a still larger bone formation rate in Gd-WH/CS scaffolds than in WH/CS.

### 3.9 Histological analysis

**FIGURE 11:**
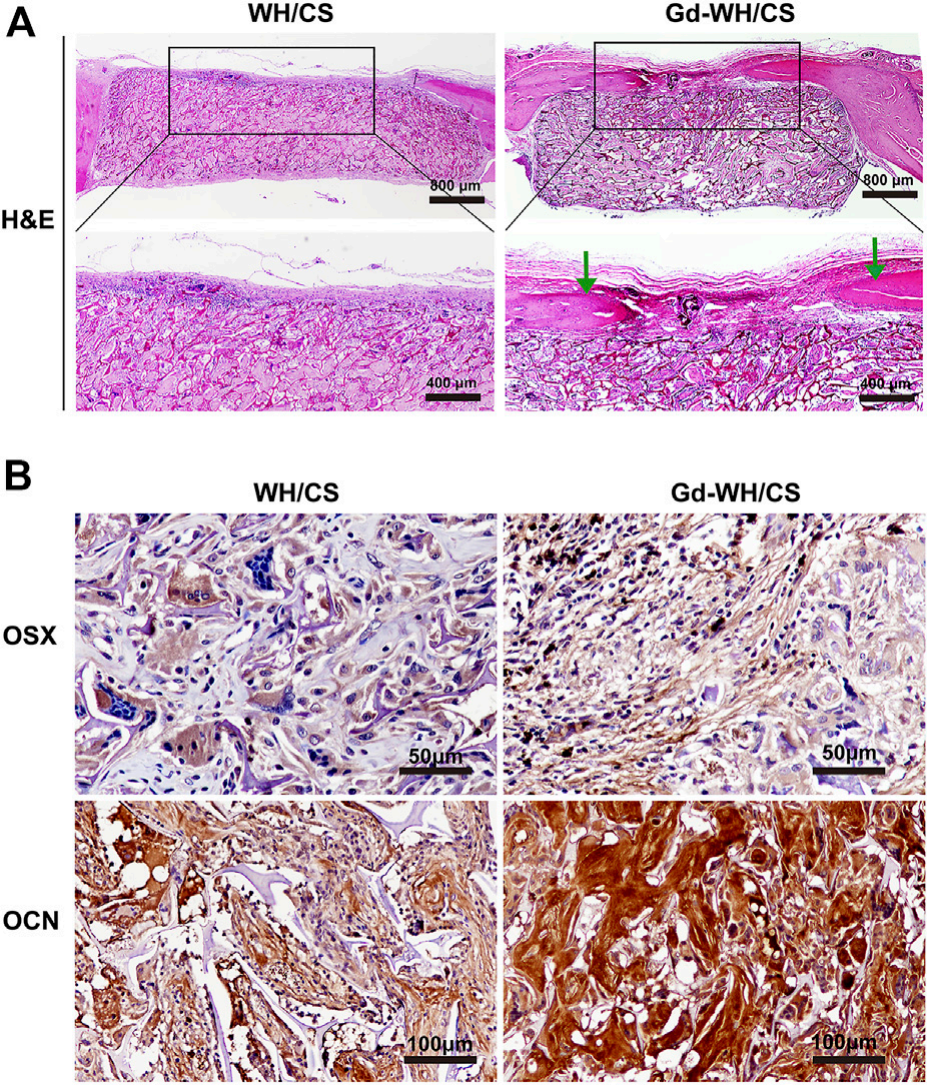
Histological analysis. **(A)** Hematoxylin and eosin (H&E) staining of the cranial defects implanted with Gd-WH/CS scaffolds and WH/CS scaffolds at 8 weeks, respectively. The green arrow indicates new bone formation. **(B)** Immunohistochemical staining of OSX and OCN. The Gd-WH/CS group demonstrated significantly increased positive staining compared with the WH/CS group.

H&E staining and immunohistochemical staining were performed to further examine the newly formed bone. As shown in Figure 11A, H&E staining of the craniums implanted with Gd-WH/CS scaffolds revealed more bone tissue in bone defect area, indicated better bone repair than those implanted with WH/CS scaffolds. In addition, the immunohistochemical staining results of OSX and OCN were similar to the above. The positive staining of osteoblast and new bone significantly increased in the Gd-WH/CS scaffolds than in the WH/CS scaffolds (Figure 11B). All the above data indicate that Gd-WH/CS scaffolds provided outstanding capacity to repair critical-sized bone defects than the WH/CS scaffolds.

## 4 Discussion

Reducing the incidence of bone defects caused by trauma and other primary diseases is an urgent task in modern society. However, current clinically applied artificial bone substitutes are costly and have insufficient osteogenic activity. Therefore, we developed Gd-WH/CS scaffolds and assessed the biocompatibility, osteoinductivity, and bone regeneration capacity of these scaffolds as bone repair materials for the treatment of calvarial defects in an SD rat model. We preliminarily confirmed the promising potential of Gd-WH/CS scaffolds as a medical biomaterial for promoting the osteogenic differentiation of hADSCs *via* the GSK3β/β-catenin signaling pathway by *in vitro* and *in vivo* evaluations. Moreover, Gd-WH/CS scaffold also has a suitable mechanical strength and stable degradation rate as a bone tissue scaffold, making it ideal for bone tissue implantation with long-term therapeutic effects.

The as-obtained Gd-WH/CS and WH/CS scaffolds by freeze-drying method possessed 3D interconnected macroporous structure, with a pore size ranging 150–300 μm (Figure 4A; Supplementary Figure S3), the porosity of CS, WH/CS and Gd-WH/CS composite scaffolds respectively were 73.47%, 84.37% and 85.08%. These macropores in the scaffolds provided 3D channels which facilitated the growth of bone precursor cells and tissues into scaffold, besides, the scaffolds have excellent mechanical properties (Supplementary Figure S4), resulting in admirable osteoconductivity. Bone tissue and cells proliferated, spread out, and migrated to the surface and interior of the entire scaffolds *via* the micropores as in-growth channels after the scaffolds were implanted into the calvarial bone defects (Figures 10A, 11A). Moreover, our *in vitro* and *in vivo* biocompatibility results showed that the scaffolds have no cytotoxic or tissue toxicity (Figures 5, 11), which demonstrate the excellent biosafety of the Gd-WH/CS and WH/CS scaffolds. The excellent biocompatibility of the scaffolds depends on the physicochemical properties of the main components of the material, which in this study are WH and CS. WH is an important inorganic component of bone tissues, and CS is a natural cationic polysaccharide-type biopolymer, both of which have excellent biocompatibility and bioactivities (Lei et al., 2017). Meanwhile, we observed that the incorporation of Gd^3+^ ions in WH nanocrystals decreased their crystallinity (Figure 1A). In general, the biological effects of REEs, whether beneficial or harmful, are concentration-dependent and may potentially cause harm to the human body at higher concentrations. Thus, we first examined the cytotoxicity and osteogenic activity of Gd^3+^ ions at low concentrations prior to *in vivo* testing. The results showed no cytotoxic effect of Gd on hADSCs when the Gd^3+^ ion concentration was <100 μM (Figure 7A), and the concentration of Gd^3+^ ions as-released from the Gd-WH/CS scaffolds arrived at 13.27 μM after soaking for 48 h (Supplementary Table S1), which was kept in the safe concentration range. Meanwhile, the ALP and ARS results showed that Gd^3+^ ions could produce osteogenic activity on hADSCs at this low concentration (Figures 7B, C). Therefore, Gd-containing scaffolds should be slowly degraded to release Gd^3+^ ions following implantation to ensure the biosafety of the bone repair therapy. Moreover, we believe that the high chemical stability (Supplementary Figure S2) of Gd-WH/CS caused the slow release of Gd^3+^ ions from the scaffolds (Supplementary Figure S1). These results confirmed that the Gd-WH/CS scaffolds have excellent biosafety, which is important for its application in clinical setting.

When ideal bone scaffolds are implanted into a bone defect site, they can produce osteogenic activity and promote bone regeneration, thereby treating bone defects to overcome the shortcomings of the above-mentioned bone substitute products. In this study, we determined the osteogenic activity of Gd-WH/CS composite scaffold by observing its bioeffect on the osteogenic differentiation of hADSCs. We observed ALP and RAS staining intensities of hADSCs were higher in Gd-WH/CS group than those in the WH/CS group (Figure 6), suggesting that the Gd ions in the scaffolds promoted the osteogenic differentiation of hADSCs (Guo et al., 2014). Wnt signaling pathway is considered to be an important pathway regulating osteogenic differentiation, its activity is mediated through GSK3β, which promotes the phosphorylation and degradation of β-catenin, initiating the osteogenic-related genes transcription (Liu et al., 2014; Zhang et al., 2016). Thus, inhibition of GSK3β indeed promoted osteogenic differentiation. Hence, we tested whether enhancing osteogenic differentiation by Gd^3+^ was associated with the inhibition of GSK3β activity. Western blot results demonstrated that Gd decreased the GSK3β and increased the β-catenin activity in a dose-dependent manner (Figure 8A). Furthermore, the expression of osteogenic-related genes, including osteocalcin (OCN), osterix (OSX), and collagen 1A1 (COL11A), was quantified using real-time PCR, the results of which indicated that the osteogenic-related genes expression level was significantly upregulated with the treatment of Gd^3+^ in a dose-dependent manner (Figure 8B). Therefore, we confirmed that one of the mechanisms for enhancement of osteogenic differentiation ability of hADSCs and regeneration of bone tissue around the Gd-WH/CS scaffold may involve the GSK3β/β-catenin signaling pathway.

By constructing a rat calvarial bone defect model, we could test the bone repair properties of Gd-WH/CS scaffolds. The bone repair effect of WH/CS scaffolds and Gd-WH/CS scaffolds was observed. Compared with WH/CS scaffolds, there was significantly more new bone formation (Figures 9A, E), more calcium depositions and bony nodules (Figure 10A) around and inside the Gd-WH/CS scaffolds. Besides, H&E staining revealed a larger degradation area of the Gd-WH/CS scaffolds than that in the WH/CS scaffolds, and correspondingly more bone tissue grew in the Gd-WH/CS scaffolds (Figure 11A). This suggests that in addition to better bone regeneration properties, the Gd-WH/CS scaffolds may have a faster bone repair cycle since its appropriate rate of degradation is more consistent with the rate at which new bone grows into the scaffold. This was attributed to the possibility that Gd^3+^ ions in the Gd-WH nanocrystals decreased the crystallinity (Figure 1), thus slightly increased the biodegradability (Supplementary Figure S2). Moreover, in the immunohistochemical staining test, OSX and OCN positive staining significantly increased in the Gd-WH/CS group compared with that in WH/CS group (Figure 11B). Which further demonstrated the positive effects of Gd ions on osteoinductivity and new bone formation (Hoang et al., 2003; Choi et al., 2016). Together, the Gd-WH/CS composite scaffolds possessed an excellent osteoinductivity for bone tissue healing. When the scaffold is implanted into the bone defect site, it will degrade at an appropriate rate, promote the bone tissue regeneration around scaffold. New bone grows and eventually replaces the scaffold completely, thus proposing a new cost-efficient treatment strategy for bone defect disease.

Our study has several limitations. First, although the biocompatibility results showed that the scaffolds are non-cytotoxic, but this conclusion is based on the assumption that the Gd^3+^ ions were almost drained from the body. However, accurate and complete metabolic pathways of Gd^3+^ ion must be determined before clinical application. Second, although the calvarial bone defect model used in this study can intuitively show the bone regeneration capacity of the scaffold, the mechanical environment of the calvarial bone region is different from that of limbs and joints. Therefore, the biomechanical performance advantages of the material cannot be accurately conveyed. In future studies, for the treatment of bone defects, we intend to use different animal models to implant scaffolds in weight-bearing areas of limbs such as the femur or tibia; this may provide valuable data for clinical applications.

## 5 Conclusion

To address some of the limitations of existing therapeutic strategies and propose a potential solution for bone defect disease, Gd-WH/CS composite scaffolds with favorable biocompatibility and biodegradability were fabricated for bone tissue engineering applications. During the *in vitro* and *in vivo* evaluations, Gd-WH/CS was demonstrated to have an excellent bone regeneration capacity and therapeutic effect in a rat calvarial bone defects model. We also preliminarily ascertained that one of the potential mechanisms for promoting osteogenic differentiation of hADSCs and regeneration of bone tissue around Gd-WH/CS scaffolds is achieved *via* the GSK3β/β-catenin signaling pathway, which also significantly upregulated the expression of osteogenic related genes. These results suggest the Gd-WH/CS composite scaffold has clinical application value as an effective repair material for the treatment of bone defects related diseases.

## Data Availability

The data that support the findings of this study are available from the corresponding authors upon reasonable request.
