# Supplementary material for: Gadolinium-doped whitlockite/chitosan composite scaffolds with osteogenic activity for bone defect treatment: In vitro and in vivo evaluations
Source: Front Bioeng Biotechnol. 2023 Feb 15;11:1071692. doi: 10.3389/fbioe.2023.1071692 (PMC9975562; doi:10.3389/fbioe.2023.1071692)
Supplement: Supplementary file 1 [file DataSheet1.PDF]

Supplemental material

Methods and Results

1. Quantitative analysis of ions releases from scaffolds

An inductively coupled plasma spectrometer (ICP-OBS, aglient 5110) was used to measure Ca, P, Mg, and Gd ion release from the scaffold material in the simulated solution. 0.4g of Gd-WH/CS scaffold material was put into 10mL of cell culture medium, and 4mL of the released solution was taken out as the detection solution at the specified time (6h, 12h, 24h, 48h, 72h, 96h, 144h), and then 4mL of new cell culture medium was added to continue ion release. The ion concentrations of Ca, P, Mg, and Gd in the Gd-WH/CS extracts were also measured.

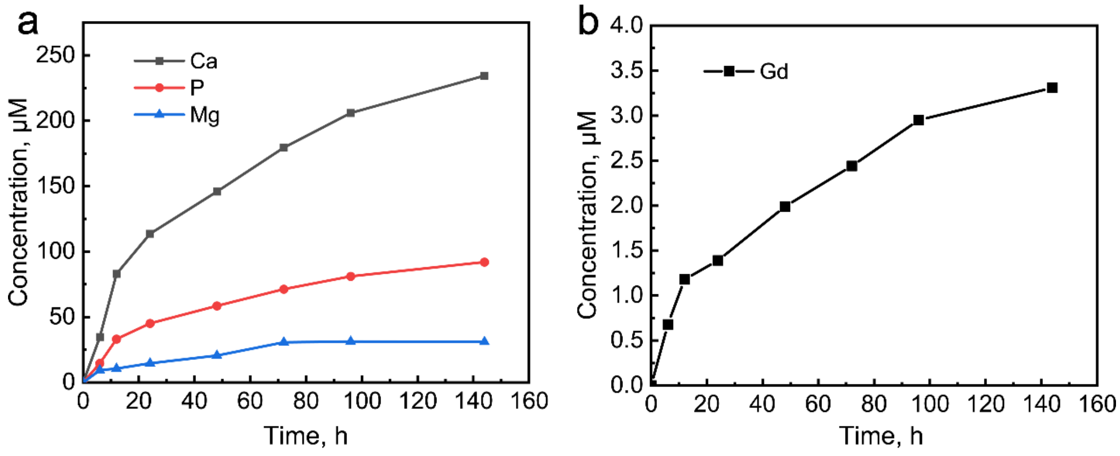

Figure. S1 Ion release profile of Gd-WH/CS scaffolds: (a) Ca, Mg, P ions; (b) Gd ion.

**Ions**	**Concentration (**mmol/L)
**Gd**	13.27
**Mg**	138.92
**Ca**	971.96
**P**	389.84

Table S1. Concentrations of Gd, Mg, Ca and P ions in the Gd-WH/CS extracts.

According to the release results (Figure. S1), Ca, Mg, P and Gd ions were released at a fast rate during the initial 24 h of release. With the extension of release

time, Gd-WH nanoparticles in the scaffold reached the dynamic equilibrium process of dissolving and precipitation. Although the total amount of release increased gradually with the extension of time, the ion release rate decreased. After 144 h of release, the amount of Gd ion released reached 3.31 μM. Moreover, the degradation rate of scaffolds has an important effect on the release rate of Gd ions. From the general trend of the release curve, the release of Gd ions is a slow-release process. In addition, the concentration of Gd ion in the extracts is about 13μM (Table S1), which has been shown to have biological effects in our *in vitro* experiments.

2. The hydrolytic and enzymatic degradation of the scaffolds

The hydrolysis and enzymatic degradation of the scaffolds were measured gravimetrically. Three groups of 0.03g CS, WH/CS and Gd-WH/CS scaffolds were placed into EP tubes of 5ml PBS solution. The initial scaffold was weighed first and the weight W0 was recorded. Then the scaffold was immersed in a constant temperature of 37 ℃ and an oscillation frequency of 80r/min in a constant temperature oscillator. At specific time intervals (2h, 4h, 6h, 12h, and 24h), the swelled samples were removed and the mass W1 was taken. All experiments were repeated three times. Then at specific time intervals (1, 2, 4, 6, 8 days), the degraded samples were removed and freeze-dried, and the mass W1 was weighed again. All experiments were repeated three times.

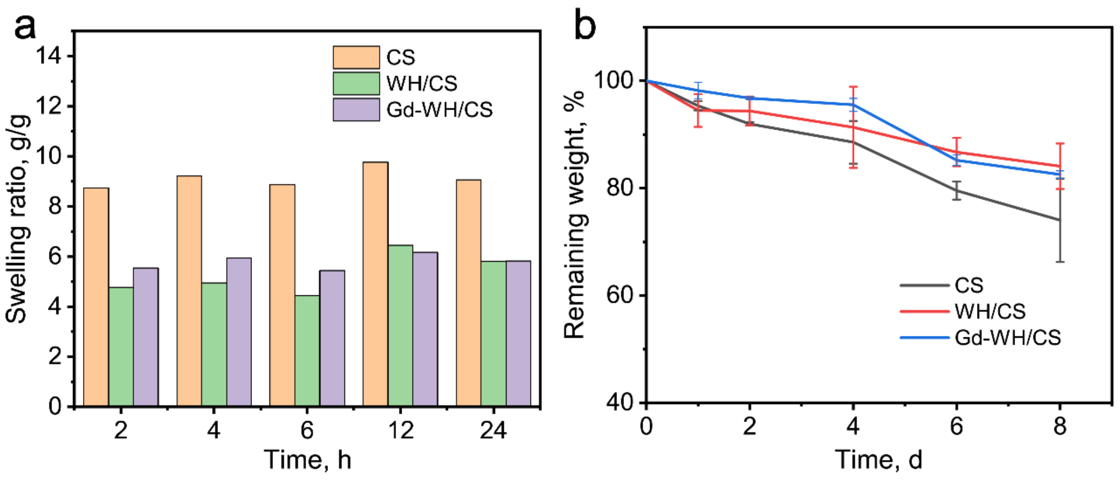

Figure. S2 (a) Swelling curves of CS, WH/CS and Gd-WH/CS composite scaffolds; (b) Degradation curves of CS, WH/CS, and Gd-WH/CS composite scaffolds.

The swelling of the chitosan composite scaffold with WH and Gd-WH particles was reduced, and the pure CS scaffold showed better swelling. However, it can be seen from the figure S2a that the swelling of the Gd-WH/CS composite scaffold is slightly higher than that of the WH/CS composite scaffold. From the degradation of 8 days, it can be seen that the pure CS scaffold degrades faster (Figure. S2b), while the chitosan composite scaffold with WH and Gd-WH particles degrades less, but the composite scaffold with Gd is slightly faster than the composite scaffold without it.

3. Porosity percentage and pore size distribution of scaffolds

The porosity of the prepared scaffold material was detected by using the liquid replacement method (Nakao et al., 2003). The calculation method satisfies the following equation:

$$P = \frac{W - W_0}{\rho V} \times 100\%$$

Among, W is the mass of the nanocomposite scaffold immersed in C_2H_5OH

solution.

V is the volume of the nanocomposite scaffold.

W_0 is the mass of the nanocomposite scaffold without immersion in C_2H_5OH solution.

ρ is the density of C_2H_5OH (20 ℃, ρ=0.790 g/mL).

The porosity of CS, WH/CS and Gd-WH/CS composite scaffolds respectively were 73.47%, 84.37% and 85.08%. And it can be seen from the figure S3 that the pore size of the Gd-WH/CS scaffold is about 195μm.

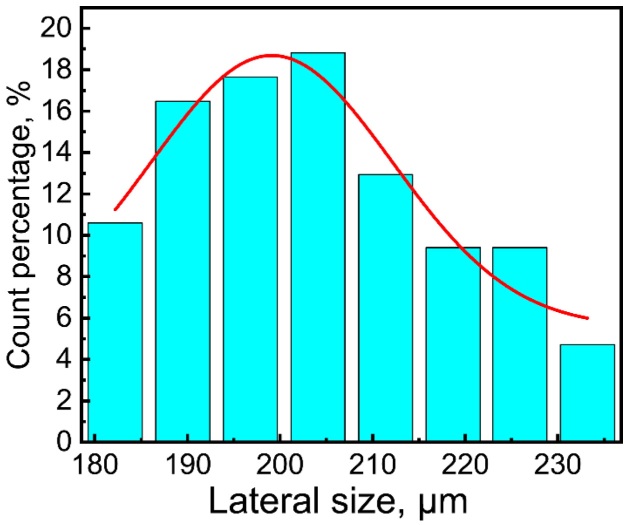

Figure. S3 Particle size histogram of Gd-WH/CS composite scaffolds.

4. Mechanical properties of the scaffolds

The compressive strength of WH/CS and Gd-WH/CS scaffolds (r = 1.3 cm, h= 1.0cm) at a compression rate of 5 mm/min was measured by a microcomputer-controlled electronic universal testing machine (WDW-0.5C, Shanghai Hualong Microelectronics Co., LTD., China).

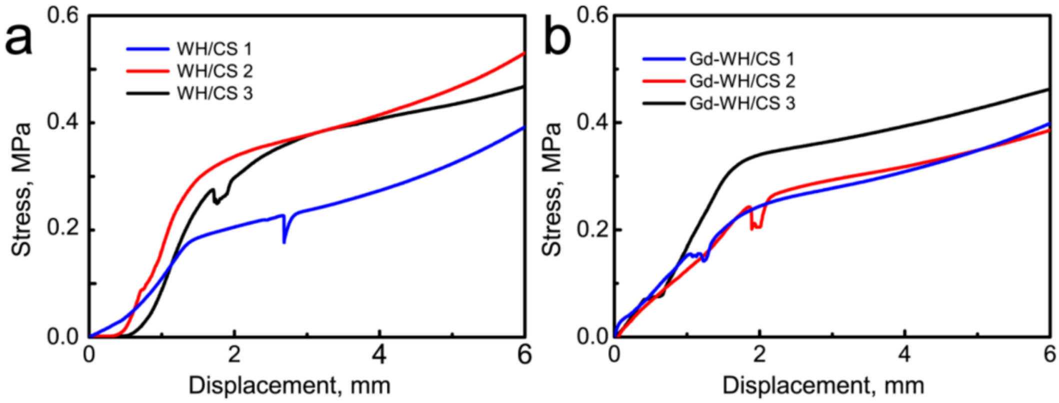

Figure. S4 Mechanical properties of the scaffold materials: (a) WH/CS composite scaffold; (b) Gd-WH/CS composite scaffold;

The test results (Figure. S4) showed that the prepared scaffolds had good mechanical properties. For the pure WH/CS composite scaffold material, after three measurements, the force used to destroy the pore structure of the scaffold material was 0.20 MPa in the first stage, and then the pressure continued to increase. Since the scaffold material contained chitosan, the scaffold material was generally plastic material, so with the increase of force, the scaffold material was gradually compressed until it reached a compacted state. For the Gd-WH/CS composite scaffold material, the force used to destroy the internal structure of the scaffold was 0.18 MPa.

5. TGA analysis of the scaffolds

In order to understand the thermal behavior of stent, the use of the differential thermal analysis (Rigaku tg-dta 8122) at room temperature to 900℃ tested in the air, heating rate in the Pt crucible for 10 ℃/min.

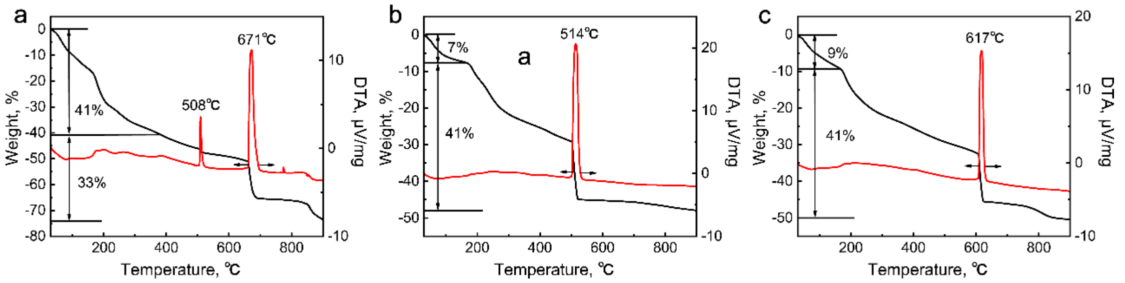

Figure. S5 TG-DTA of different scaffolds: (a) CS; (b) HA/CS; (c)Gd-WH/CS

The figure S5 shows the loss process of CS from 30 ° C to 900 ° C. The weight loss process can be mainly divided into two parts, the first part occurs from 30 to 380 ° C, and the weight loss is about 41%. This can be attributed to the physical adsorption of water in the CS scaffold and the evaporation of crystalline water. The second part mainly occurs between 380 ° C and 900 ° C, and the weight loss is about 33%, which can be attributed to the degradation of CS with the formation of a new phase, and the accompanying heating process, with an exothermic peak of 508 ° C. Loss process of WH/CS at 30 ° C to 900 ° C. The weight loss process is again divided into two parts, the first part mainly occurs from 30 to 170 ° C, and the weight loss is about 7%, which is mainly attributed to the physical adsorption of water in the composite scaffold and the evaporation of crystalline water. The second part mainly occurred at 170℃ to 900℃. Since WH was stable at 170℃ to 900℃, the weight lost was mainly caused by the decomposition of CS, indicating that the mass of CS accounted for about 41% of the scaffold mass. Loss process of Gd-WH/CS at 30 ° C to 900 ° C. The weight loss process is again divided into two parts, the first part mainly occurs from 30 to 170 ° C, and the weight loss is about 9%, which is mainly attributed to the evaporation of water physically adsorbed and crystalline water in the composite scaffold. The second part mainly occurred at 170℃ to 900℃. Since WH

was stable at 170℃ to 900℃, the weight lost was mainly caused by the decomposition of CS, indicating that the mass of CS accounted for about 41% of the scaffold mass.

Reference

Nakao, H., Hyon, S.H., Tsutsumi, S., Matsumoto, T., and Takahashi, J. (2003). Control of pore size in L-lactide/epsilon-caprolactone copolymer foams for tissue regeneration by the freeze-drying method. *Dent Mater J* 22(3)**,** 262-271. doi: 10.4012/dmj.22.262.
